# The importance of influenza vaccination during the COVID‐19 pandemic

**DOI:** 10.1111/irv.12917

**Published:** 2021-10-03

**Authors:** John McCauley, Ian G. Barr, Terry Nolan, Theodore Tsai, Steven Rockman, Beverly Taylor

**Affiliations:** ^1^ Worldwide Influenza Centre The Francis Crick Institute London UK; ^2^ WHO Collaborating Centre for Reference and Research on Influenza VIDRL Melbourne Victoria Australia; ^3^ Department of Microbiology and Immunology, University of Melbourne Peter Doherty Institute for Infection and Immunity Melbourne Australia; ^4^ Vaccine and Immunisation Research Group Peter Doherty Institute for Infection and Immunity Melbourne Victoria Australia; ^5^ Department of Infectious Diseases, Melbourne Medical School The University of Melbourne Parkville Victoria Australia; ^6^ Vaccine and Immunisation Research Group Murdoch Children's Research Institute Parkville Victoria Australia; ^7^ Takeda Vaccines Cambridge Massachusetts USA; ^8^ Seqirus Ltd Parkville Victoria Australia; ^9^ Seqirus Ltd Liverpool UK

**Keywords:** coronavirus, COVID‐19, influenza, SARS‐CoV‐2, vaccination

## Abstract

The COVID‐19 pandemic and the measures taken to mitigate its spread have had a dramatic effect on the circulation patterns of other respiratory viruses, most especially influenza viruses. Since April 2020, the global circulation of influenza has been markedly reduced; however, it is still present in a number of different countries and could pose a renewed threat in the upcoming Northern Hemisphere winter. Influenza vaccination remains the most effective preventive measure that we have at our disposal against influenza infections and should not be ignored for the 2021–2022 season.

As the 2021 Northern Hemisphere (NH) winter approaches, once again there will be a call for people to be vaccinated against influenza. In 2020, with high levels of COVID‐19 infections and strict public health and social interventions dominating in many countries, little or no influenza circulated.^1^, ^2^ In the second half of 2021, as countries approach high levels of immunity to COVID‐19 through vaccination and/or infection, local and international travels have and will inevitably increase. Enhanced human movement is likely to increase the risk of introduction and spread of influenza and other respiratory diseases from countries where these viruses are circulating. Beyond 2021, the reduction in influenza exposure may increase the population's susceptibility to new influenza variants even further, leading to more extensive outbreaks of influenza. The Joint Committee on Vaccination and Immunisation (JCVI) in the United Kingdom has recommended a synergistic programme of vaccination for flu and COVID‐19 in preparation for this possibility.^3^ This commentary explores the need for influenza vaccination taking into account the influence of COVID‐19 infection and vaccination.

The COVID‐19 pandemic has had an unprecedented impact on humans and society and thereby on the dynamics of human infectious disease, particularly influenza. Non‐pharmaceutical interventions (NPIs) resulted in reduced local and international travels, school closures, social distancing and possibly as seen with other respiratory viruses, virus interference,^4^ all of which have played a major part in reducing the circulation of human influenza viruses. GISRS (the WHO Global Influenza Surveillance and Response System) monitors worldwide influenza circulation through 147 WHO National Influenza Centres in 126 countries or territories. These laboratories report their data to WHO through two internet platforms both of which are publicly accessible: FluID and FluNet, which collate influenza epidemiological data and the number of influenza viruses detected by virus type and subtype on a weekly basis by country and region—these data are critical for tracking influenza activity and the movement of viruses globally.

WHO reported that influenza activity between September 2020 and January 2021 was mostly present in countries in the tropics and subtropics but was also reported in some countries in the temperate zone of the NH.^5^ Of these samples, influenza viruses tested positive in less than 0.2% of the total samples, contrasting sharply with an average of 17% over the previous three seasons prior to the emergence of COVID‐19. Nevertheless, new human influenza viruses were still detected in this period. Influenza A(H3N2) viruses emerged in Cambodia and Bangladesh and subsequently spread to other countries, resulting in an update to the composition of the influenza vaccines to be used in the NH 2021/2022 influenza season. Furthermore, antigenically distinct influenza B viruses were detected in China, in parts of West Africa and elsewhere. While influenza circulation has continued at lower levels since the start of 2021, 45 countries have reported 10 or more influenza detections from their sentinel surveillance systems, indicating influenza circulation in that country, albeit limited (Figure 1). At the time of writing, influenza virus circulation in the Southern Hemisphere winter influenza season was still very low, making it difficult to predict which viruses will emerge in late 2021 and early 2022.

**FIGURE 1:**
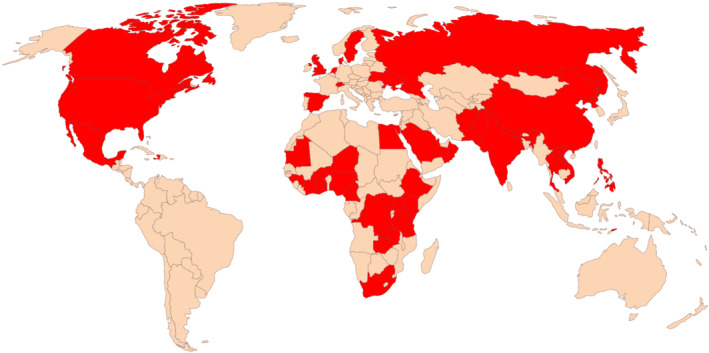
Countries with >10 lab confirmed influenza cases (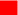) reported to WHO FluNet in 2021 (https://www.who.int/tools/flunet up to 17 September 2021) (n = 45) (world map by www.freeworldmaps.net)

As COVID infections decrease and vaccinations increase worldwide with an accompanying easing of restrictions, there is an opportunity for a resurgence of respiratory viruses to occur. An important lesson of how quickly the resurgence of a respiratory disease can arise was seen recently in Australia with respiratory syncytial virus (RSV). Normally, in the temperate regions of Australia, the seasonality of RSV has been very predictable, with cases rising in April–May each year, peaking in the winter months of June–July, before subsiding in August–September.^6^ However, in 2020, the RSV season was atypical, with just a few sporadic cases, but this changed dramatically in late 2020 when a large outbreak occurred in New South Wales, shortly followed by outbreaks in Western Australia, Victoria and Queensland.^7^ These outbreaks were very unusual as they occurred in the Spring–Summer period and, significantly, were as severe as those in a normal season, with many paediatric hospitalizations along with adult infections. There has also been a recent outbreak of RSV in the South of France^8^ after virtually no RSV cases since late 2019–2020 with similar delayed outbreaks also seen in the United States.^9^ While there are differences in the demographics and the global circulation of RSV and influenza, the two share many similarities including the groups that are most vulnerable to infection, the elderly and the very young. Fortunately for influenza, both prophylactic vaccines and antiviral drugs are available, whereas for RSV, there are limited options for the prevention or treatment of disease.

Despite the uncharacteristic decline in influenza circulation worldwide, there is every likelihood that influenza will re‐emerge and continue to affect humans at similar levels to that of the past. Low levels of influenza virus transmission in 2020 limited the opportunities to observe clinical features of dual SARS‐CoV‐2 and seasonal influenza virus infections.^10^ Of the few reports, some suggest no difference in the clinical course of dually infected patients compared with those infected with SARS‐CoV‐2 alone; however, both improved and worse outcomes have been reported.^11^, ^12^, ^13^, ^14^ From a clinical and public health perspective, the burden on healthcare systems due to epidemics of either virus alone, let alone overlapping epidemics, remains a credible threat. Thus, without intervention, the potential of a ‘syndemic’—a term coined to describe twin synergistic epidemics—due to the cocirculation of influenza and COVID‐19 is a serious reality, especially as public health and social measures are relaxed. The presence of two respiratory infections that might not be distinguishable by clinical signs is also important for disease management. Correctly identifying the infecting virus is critical for guidance with respect to treatment, isolation and quarantine.

The US CDC has recently recommended that routine vaccines could be coadministered with authorized COVID‐19 vaccines, in order to facilitate the catch‐up of missed immunizations.^15^ This public health decision was not based on new clinical trial evidence but on the accumulated safety experience of the currently authorized COVID‐19 vaccines in millions of recipients, albeit over a relatively short time frame, and the previous experience of safe and effective administration of multiple antigens simultaneously.^16^ Safety data on the coadministration of influenza and COVID‐19 vaccines are currently being acquired. In the United Kingdom, the JCVI has stated that early evidence on the concomitant administration of COVID‐19 and influenza vaccines supports the delivery of both vaccines at the same time where appropriate and that this approach may help to maximize uptake of both vaccines.^3^

One such study carried out as part of a substudy on Seqirus's Flucelvax Quadrivalent and Fluad trivalent vaccines coadministered with the Novavax's COVID‐19 vaccine (NVX‐CoV2373) as part of their Phase 3 randomized trial showed that coadministration resulted in no change to influenza vaccine immune responses and only a slight reduction in the NVX‐CoV2372 immune response.^17^ The ComFluCOV clinical trial to assess the safety and immune responses generated when giving COVID‐19 and influenza vaccines has reported that all study participants have had their second visit for vaccination and so results are eagerly awaited (https://comflucov.blogs.bristol.ac.uk/). For the Southern Hemisphere season, Australian authorities are currently recommending a minimum of 7 days (previously 14 days) between COVID‐19 vaccines and influenza vaccines (or vice versa) unless there are extenuating circumstances. As COVID‐19 vaccines are further studied and potentially authorized for young children and infants, careful consideration and evidence for safe and effective coadministration with influenza and other routine vaccines is in children also warranted.^18^ As the available data to date indicate that coadministration of vaccines is a viable approach, there is benefit in continuing to generate more data to support this as it would facilitate the catch‐up of missed vaccinations and would also expedite an efficient outcome for dual protection against influenza and COVID‐19.

In summary, even though influenza circulation is currently low globally, influenza viruses are still in circulation and can be rapidly transported when air travel returns leading to increased infections and potentially epidemics in late 2021 or 2022 as predicted in a recent modelling study.^19^ Hence, as the threat of influenza is still present and now more likely to return, influenza vaccination remains the best way to mitigate against infection. So roll up your sleeves once more, just to be sure!

## AUTHOR CONTRIBUTIONS

**John McCauley:** Conceptualization. **Ian G. Barr:** Conceptualization; data curation. **Terry Nolan:** Writing and editing. **Theodore Tsai:** Writing and editing. **Steven Rockman:** Conceptualization. **Beverly Taylor:** Conceptualization.

### PEER REVIEW

The peer review history for this article is available at https://publons.com/publon/10.1111/irv.12917.

## Data Availability

Data are derived from public domain resources.
